# A multivariate genetic analysis of anxiety sensitivity, environmental sensitivity and reported life events in adolescents

**DOI:** 10.1111/jcpp.13725

**Published:** 2022-12-13

**Authors:** Alicia J. Peel, Olakunle Oginni, Elham Assary, Georgina Krebs, Celestine Lockhart, Thomas McGregor, Elisavet Palaiologou, Angelica Ronald, Andrea Danese, Thalia C. Eley

**Affiliations:** ^1^ Social, Genetic and Developmental Psychiatry Centre Institute of Psychiatry, Psychology and Neuroscience, King's College London London UK; ^2^ Research Department of Clinical, Educational and Health Psychology University College London London UK; ^3^ National and Specialist OCD, BDD and Related Disorders Clinic for Young People South London and Maudsley NHS Foundation Trust London UK; ^4^ Centre for Brain and Cognitive Development, Department of Psychological Sciences Birkbeck, University of London London UK; ^5^ Department of Child & Adolescent Psychiatry Institute of Psychiatry, Psychology & Neuroscience, King's College London London UK; ^6^ National and Specialist CAMHS Trauma, Anxiety, and Depression Clinic, South London and Maudsley NHS Foundation Trust London UK; ^7^ NIHR Biomedical Research Centre, South London and Maudsley Hospital London UK

## Abstract

**Background:**

Despite being considered a measure of environmental risk, reported life events are partly heritable. One mechanism that may contribute to this heritability is genetic influences on sensitivity, relating to how individuals process and interpret internal and external signals. The aim of this study was to explore the genetic and environmental overlap between self‐reported life events and measures of sensitivity.

**Methods:**

At age 17, 2,939 individuals from the Twins Early Development Study (TEDS) completed measures of anxiety sensitivity (Children's Anxiety Sensitivity Index), environmental sensitivity (Highly Sensitive Child Scale) and reported their experience of 20 recent life events. Using multivariate Cholesky decomposition models, we investigated the shared genetic and environmental influences on the associations between these measures of sensitivity and the number of reported life events, as well as both negative and positive ratings of life events.

**Results:**

The majority of the associations between anxiety sensitivity, environmental sensitivity and reported life events were explained by shared genetic influences (60%–75%), with the remainder explained by nonshared environmental influences (25%–40%). Environmental sensitivity showed comparable genetic correlations with both negative and positive ratings of life events (*r*_A_ = .21 and .15), anxiety sensitivity only showed a significant genetic correlation with negative ratings of life events (*r*_A_ = .33). Approximately 10% of the genetic influences on reported life events were accounted for by influences shared with anxiety sensitivity and environmental sensitivity.

**Conclusion:**

Differences in how individuals process the contextual aspects of the environment or interpret their own physical and emotional response to environmental stimuli may be one mechanism through which genetic liability influences the subjective experience of life events.

## Introduction

Despite being viewed as a measure of environmental risk, self‐reported life events have been found to have a significant genetic component, with an estimated heritability of ~30% (Kendler & Baker, 2007; Power et al., 2013). Little is known about what comprises this heritability, but it is likely to reflect genetic influences on many related cognitive, behavioural and psychiatric traits. These influences may impact exposure to certain environments (gene–environment correlation) or affect the experience, interpretation or outcome of an event (gene–environment interaction). Knowledge of these mechanisms can increase understanding of the risk for negative outcomes associated with self‐reported life events. This is particularly relevant in adolescence, due to the rapid increase in major life events relating to education, puberty and social pressures during this period (Grant, Compas, Thurm, McMahon, & Gipson, 2004).

However, significant gaps remain in our understanding of how genetic factors influence self‐reports of environmental experiences. Much of the past research has been focussed on specific outcomes or disorders associated with life events, such as depression (Leighton, Botto, Silva, Jiménez, & Luyten, 2017). Taking a broader transdiagnostic approach may have greater potential to elucidate the genetic influences that interact with the environment, rather than just the components that are shared with certain disorders. One set of traits that are hypothesised to influence the transdiagnostic outcomes of environmental experiences are sensitivity biases (Pluess, 2015).

Sensitivity relates to how individuals process and interpret internal and external signals. One example is anxiety sensitivity, the enhanced awareness of the symptoms of anxiety, such as heart palpitations or worry, and tendency to perceive these as being harmful (Taylor, 2014). Anxiety sensitivity is moderately heritable, with estimates ranging from 37% in children (Eley, Gregory, Clark, & Ehlers, 2007) to 45% in adolescents (Stein, Jang, & Livesley, 1999; Zavos, Rijsdijk, Gregory, & Eley, 2010). In young adults, anxiety sensitivity has been shown to influence the association between stressful events and later symptoms of anxiety (McLaughlin & Hatzenbuehler, 2009) and posttraumatic stress (Feldner, Lewis, Leen‐Feldner, Schnurr, & Zvolensky, 2006). It has also been associated with increased risk of suicidal ideation among individuals undergoing stressful experiences (Capron, Cougle, Ribeiro, Joiner, & Schmidt, 2012). These findings indicate that propensity towards anxiety sensitivity may interact with the experience of stressful events to exacerbate their impact on psychopathology.

However, sensitivity biases may not only influence the impact of *adverse* events. Under the differential susceptibility model, genetic predispositions do not only confer ‘vulnerability’ to negative experiences, but rather broader ‘sensitivity’ to the environment generally (Belsky et al., 2009). General sensitivity to the environment is proposed to moderate both the adverse effects of negative experiences as well as the tendency to benefit from positive environments (Belsky & Pluess, 2009). Using measures that capture the thoughts and behaviours of sensitive individuals, genetic influences were found to account for 47% of the variance in environmental sensitivity in adolescents (Assary, Zavos, Krapohl, Keers, & Pluess, 2021). In support of the differential susceptibility model, high environmental sensitivity has been associated with both increases in mood and wellbeing following positive life events (Iimura, 2021) and decreases following stressful exposures (Pluess, Lionetti, Aron, & Aron, 2020).

In young adults, high environmental sensitivity is associated with greater perceived stress, but not differences in physiological measures of arousal (Benham, 2006; Weyn et al., 2022). One explanation for this is that highly sensitive individuals may be more aware of their own physiological responses and may notice minor sensations that less sensitive individuals would not (Benham, 2006). This would suggest that environmental sensitivity is subtly different from anxiety sensitivity. The former relates to responses to the environment while the latter relates to the pathological misinterpretation of these responses as harmful. It may also indicate that both environmental and anxiety sensitivity are related to increased awareness of physiological symptoms and, therefore, may share underlying genetic influences.

However, there have been no previous investigations of whether these two types of sensitivity are phenotypically and genetically related. Additionally, there is limited understanding of the extent to which these traits contribute to the genetic underpinnings of self‐reported life events. This knowledge would inform understanding of what is captured by the heritable component of self‐reported experiences. If sensitivity biases are associated with reported life events, they may represent modifiable, transdiagnostic targets for intervening in the negative outcomes of adverse environments. Relating to these gaps in knowledge, this study aimed to explore the shared genetic basis of anxiety sensitivity, environmental sensitivity and reported life events. We hypothesised that:

1. Anxiety sensitivity, environmental sensitivity and reported life events would display moderate genetic correlations.
2. Environmental sensitivity would display positive genetic correlations with both positive and negative life events, whereas anxiety sensitivity would only be positively correlated with negative life events.
3. A moderate proportion of the genetic influences on reported life events would be shared with the influences on anxiety sensitivity and environmental sensitivity.

## Methods

All analyses were preregistered on the Open Science Framework (OSF) prior to accessing the data (https://osf.io/haud4/). Custom code for these analyses is available on the OSF website.

### Sample

Data for these analyses were drawn from the Twins Early Development Study (TEDS), a study of over 15,000 twin pairs born in England and Wales between 1994–1996 (Rimfeld et al., 2019).

Between ages 15–17 years, 5,163 families returned data from the Longitudinal Experiences And Perceptions (LEAP) wave of data collection. A subset of 1,773 families were then invited to participate in the LEAP‐2 follow‐up study approximately 9 months later, which included measures of sensitivity and life events. The full LEAP‐2 study booklet is accessible through the TEDS data dictionary (http://www.teds.ac.uk/datadictionary). Comparison of families who did and did not take part in LEAP‐2 is given in Table S1. Individuals who completed at least one of the measures of anxiety sensitivity, environmental sensitivity or reported life events in LEAP‐2 formed the study sample (~500 monozygotic twin pairs and ~900 dizygotic twin pairs). In accordance with standard exclusion criteria for TEDS analyses, participants with severe medical disorders, who experienced severe perinatal complications, or with unknown demographic variables or zygosity were excluded (https://www.teds.ac.uk/datadictionary/exclusions.htm). This resulted in a final sample of 2,939 individuals (59% female) with an average age of 17.1 years (*SD* = 0.9).

### Ethical considerations

Ethical approval for TEDS was provided by the King's College London Ethics Committee (reference: PNM/09/10–104). Written informed consent was obtained from parents and twins prior to data collection.

### Measures

#### Anxiety sensitivity

Anxiety sensitivity was assessed using the Children's Anxiety Sensitivity Index (CASI; Silverman, Fleisig, Rabian, & Peterson, 1991). The CASI is an 18‐item self‐report questionnaire that asks participants whether statements capturing fear of anxiety sensations are *Not true* (0), *Quite true* (1) or *Very true* (2) over the last 6 months (Table S2). The internal consistency of the CASI in TEDS is *α* = .93 (Eley et al., 2007). Responses were summed to give total scores, with higher scores representing higher levels of anxiety sensitivity.

#### Environmental sensitivity

Environmental sensitivity was assessed with the 12‐item Highly Sensitive Child (HSC) scale, developed to capture the typical behaviours and experiences of sensitive children and adolescents (Pluess et al., 2018). The HSC scale assesses three domains of environmental sensitivity related to becoming mentally overwhelmed by contextual stimuli, awareness of aesthetic details and unpleasant reactivity to sensory stimuli (Table S3). Participants are asked to rate the extent to which each statement describes them, on a Likert scale ranging from *Not at all* (1) to *Extremely* (7). The internal consistency of the scale in TEDS is *α* = .81 (Assary et al., 2021). Responses were summed to give total scores, with higher scores representing higher levels of environmental sensitivity.

#### Reported life events

Reported life events were assessed using a reduced version of the Coddington Life Events Scale (Coddington, 1972), comprising the 20 items most relevant to adolescents. Participants were asked to report whether they had experienced any of the events in the past 6 months and whether they found the experience ‘Very unpleasant’, ‘Moderately unpleasant’, ‘Neither unpleasant nor pleasant’, ‘Moderately pleasant’ or ‘Very pleasant’ (Table S4). A breakdown of the proportion of responses for each event is given in Table S5.

This measure was used to derive three variables (Table S6). First, the total number of reported life events was counted. All responses indicating the experience of an event (‘Yes’, ‘Very unpleasant’, ‘Moderately unpleasant’, ‘Neither unpleasant nor pleasant’, ‘Moderately pleasant’ and ‘Very pleasant’) were collapsed into one category representing ‘Present’ (1), while the response option ‘No’ represented ‘Absent’ (0). Present events were summed to calculate the total number of reported life events, with scores ranging from 0 to 20. This variable was used in Models 1 and 3. For Model 2, variables representing positive and negative ratings of events were created. Positive ratings of events were calculated by summing ‘Moderately pleasant’ (1) and ‘Very pleasant’ (2) responses. Negative ratings of events were calculated by summing ‘Moderately unpleasant’ (1) and ‘Very unpleasant’ (2) responses. The responses ‘No’ and ‘Neither unpleasant nor pleasant’ were coded as 0 for both variables. In this way, whether an event is positive or negative was determined individually for each twin, based upon their own ratings. As such, items contributing to positive ratings of events for one individual could contribute to negative ratings for another. Scores for positive and negative ratings of events ranged from 0 to 40.

### Analyses

#### Descriptive statistics

Descriptive statistics including means, standard deviation and skewness were assessed for all variables. Phenotypic correlations were estimated in the full sample and in males and females separately. Cross‐twin correlations were estimated for monozygotic (MZ) and dizygotic (DZ) twins.

#### Genetic analyses

Using model fitting of twin data, the contribution of genetic and environmental influences to individual differences in a trait can be estimated (Knopik, Neiderhiser, DeFries, & Plomin, 2017). MZ twin pairs share 100% of their genes, whilst DZ twins share on average 50%. Assuming that both types of twins share their environments to similar extents, a greater degree of similarity in a trait between MZ twin pairs compared to DZ twin pairs reflects genetic influences (A). When correlations between DZ twin pairs are more than half of those between MZ twin pairs, similarity reflects shared environmental influences (C). Differences between MZ twin pairs are used to infer nonshared environmental influences (E), which also include any measurement error. In multivariate models, these principles can be applied to estimate the aetiology of the associations between traits, using cross‐twin cross‐trait correlations. Higher cross‐twin cross‐trait correlations for MZ twins compared to DZ twins indicates that covariance between two traits can be attributed to genetic influences.

To prevent inflation of the correlation between twins, variables were adjusted for age and sex, by regressing each variable on both covariates and using the residuals in subsequent analyses (McGue & Bouchard, 1984). For measures with skewness scores >1, residuals were mapped onto a normal distribution using the rank‐based van der Waerden's transformation. Genetic model fitting was conducted within R (R Core Team, 2022) using the structural equation modelling package OpenMx (Neale et al., 2016). To account for variations in sample sizes across the three measures, models were fitted to the data using Full Information Maximum Likelihood, which enables the estimation of variance components and confidence intervals in analyses with missing data, assuming that data is missing at random (Newman, 2014).

Univariate analyses were first conducted to assess the genetic, shared environmental and nonshared environmental influences on each variable (Table S7). Multivariate genetic analyses were then conducted in three stages, corresponding to each hypothesis. First, the Cholesky decomposition, interpreted as a multivariate correlated factors solution, was used to examine the shared genetic and environmental influences between anxiety sensitivity, environmental sensitivity and the total number of reported life events (Model 1). This was to test the hypothesis that these traits would share genetic influences.

Second, this model was extended by separating reported life events into negative and positive ratings, to explore their differential associations with anxiety sensitivity and environmental sensitivity (Model 2). This was to assess whether influences on the two types of sensitivity differentially overlap with the reporting of negative and positive events.

Finally, a variation of the trivariate Cholesky model was used to investigate the proportion of genetic and environmental influences on reported life events shared with anxiety sensitivity and environmental sensitivity (Model 3). In this model, the associations between anxiety sensitivity and environmental sensitivity were represented using a correlated factors solution so no direction of effect between these measures was inferred. In contrast, the genetic and environmental associations between the sensitivity measures and life events were interpreted using Cholesky decomposition paths, allowing us to account for the genetic and environmental influences on sensitivity which were shared with life events. This was to test the hypothesis that a proportion of the heritability of life events would be shared with genetic influences on sensitivity.

To facilitate multivariate genetic model fitting, means, variances and within‐person correlations were constrained to be equal across zygosity and birth order, and cross‐twin correlations were constrained to be symmetrical. To test equality of means, variances and correlations, models in which these constraints were specified were compared to corresponding saturated models in which these parameters were freely estimated. Variances and covariance were passed into A, C and E components (ACE models). For models which included small and nonsignificant estimates of C, we assessed the fit of the more parsimonious AE submodel. Model comparisons were based on likelihood ratio testing using χ^2^ values and degrees of freedom (Kline, 2015).

## Results

### Descriptive statistics

Descriptive statistics for all variables in the study sample are presented in Table 1. After adjusting for age and sex, skewness >1 persisted for all variables except environmental sensitivity, hence these variables were transformed. Phenotypic correlations for the full sample are given in Table 2 and were similar for males and females (Table S8). Cross‐twin correlations for the transformed variables were higher for MZ twins than DZ twins, indicating the influence of genetic factors. Of note, the DZ correlations were less than half of the MZ correlations for anxiety sensitivity, environmental sensitivity and negative ratings of life events, suggesting that estimates of A may include some nonadditive genetic effects. We retained the ACE specification based on previous evidence that models specifying nonadditive genetic effects (D) were not a better fit to the data for either anxiety sensitivity (Zavos, Gregory, & Eley, 2012) or environmental sensitivity (Assary et al., 2021).

**Table 1:** Descriptive statistics and intraclass twin correlations (*n* = 2,939)

Measure	Raw data		Variables regressed on age and sex	Transformed variables		
	*N*	Mean (*SD*)	Skew	Skew	Cross‐twin correlations	
					*r*_MZ_ (95% CI)	*r*_DZ_ (95% CI)
Anxiety sensitivity	2,862	8.3 (6.4)	1.08	0.00	.50 (0.44–0.56)	.15 (0.09–0.22)
Environmental sensitivity	2,799	35.8 (11.3)	0.01	–	.49 (0.42–0.55)	.22 (0.15–0.28)
Number of life events	2,584	1.7 (1.6)	2.49	0.00	.50 (0.43–0.56)	.28 (0.21–0.35)
Negative ratings of life events	2,471	1.2 (1.6)	2.14	0.00	.47 (0.40–0.54)	.21 (0.14–0.28)
Positive ratings of life events	2,471	1.2 (1.5)	1.29	0.00	.40 (0.32–0.48)	.27 (0.20–0.33)

**Table 2:** Multivariate results: standardised variance components for each measure, cross‐twin cross‐trait correlations and phenotypic correlations between measures of sensitivity and life events with proportions of variance explained by A and E

Measures	Standardised variance components (95% CI)	Associations with sensitivity measures (95% CI)					
		Anxiety sensitivity			Environmental sensitivity		
		Cross‐twin cross‐trait correlations	Phenotypic correlations	Proportion of *r*_ph_ explained by A and E	Cross‐twin cross‐trait correlations	Phenotypic correlations	Proportion of *r*_ph_ explained by A and E
Anxiety sensitivity	*h* ^2^: .47 (.40–.52) *e* ^2^: .53 (.48–.60)	–		–	–	–	
Environmental sensitivity	*h* ^2^: .48 (.42–.54) *e* ^2^: .52 (.46–.58)	*r*_MZ_: .37 (.32 to .42) *r*_DZ_: .14 (.08 to .19)	*r*_ph_: .59 (.57–.62)	A: 60% (52 to 67%) E: 40% (33 to 48%)	–	–	
Number of life events	*h* ^2^: .51 (.45–.57) *e* ^2^: .49 (.43–.55)	*r*_MZ_: .16 (.10–.21) *r*_DZ_: .04 (−.01 to .09)	*r*_ph_: .21 (.17–.25)	A: 70% (49 to 89%) E: 30% (11 to 51%)	*r*_MZ_: .12 (.07 to .17) *r*_DZ_: .02 (−.03 to .07)	*r*_ph_: .15 (.11–.19)	A: 69% (41 to 96%) E: 31% (4 to 59%)
Negative ratings of life events	*h* ^2^: .46 (.39–.52) *e* ^2^: .54 (.48–.61)	*r*_MZ_: .18 (.12 to .23) *r*_DZ_: .03 (−.02 to .08)	*r*_ph_: .20 (.16–.24)	A: 75% (53 to 97%) E: 25% (3 to 47%)	*r*_MZ_: .12 (.06 to .18) *r*_DZ_: .01 (−.04 to .06)	*r*_ph_: .14 (.10–.18)	A: 72% (38 to 104%) E: 28% (−4 to 62%)
Positive ratings of life events	*h* ^2^: .43 (.36–.49) *e* ^2^: .57 (.51–.64)	*r*_MZ_: .06 (.01 to .12) *r*_DZ_: .00 (−.05 to .05)	*r*_ph_: .09 (.05–.13)	A: 59% (−1 to 111%) E: 41% (−11 to 101%)	*r*_MZ_: .06 (.01 to .12) *r*_DZ_: .03 (−.02 to .08)	*r*_ph_: .10 (.05–.14)	A: 67% (16 to 116%) E: 33% (−16 to 85%)

**Figure 1:**
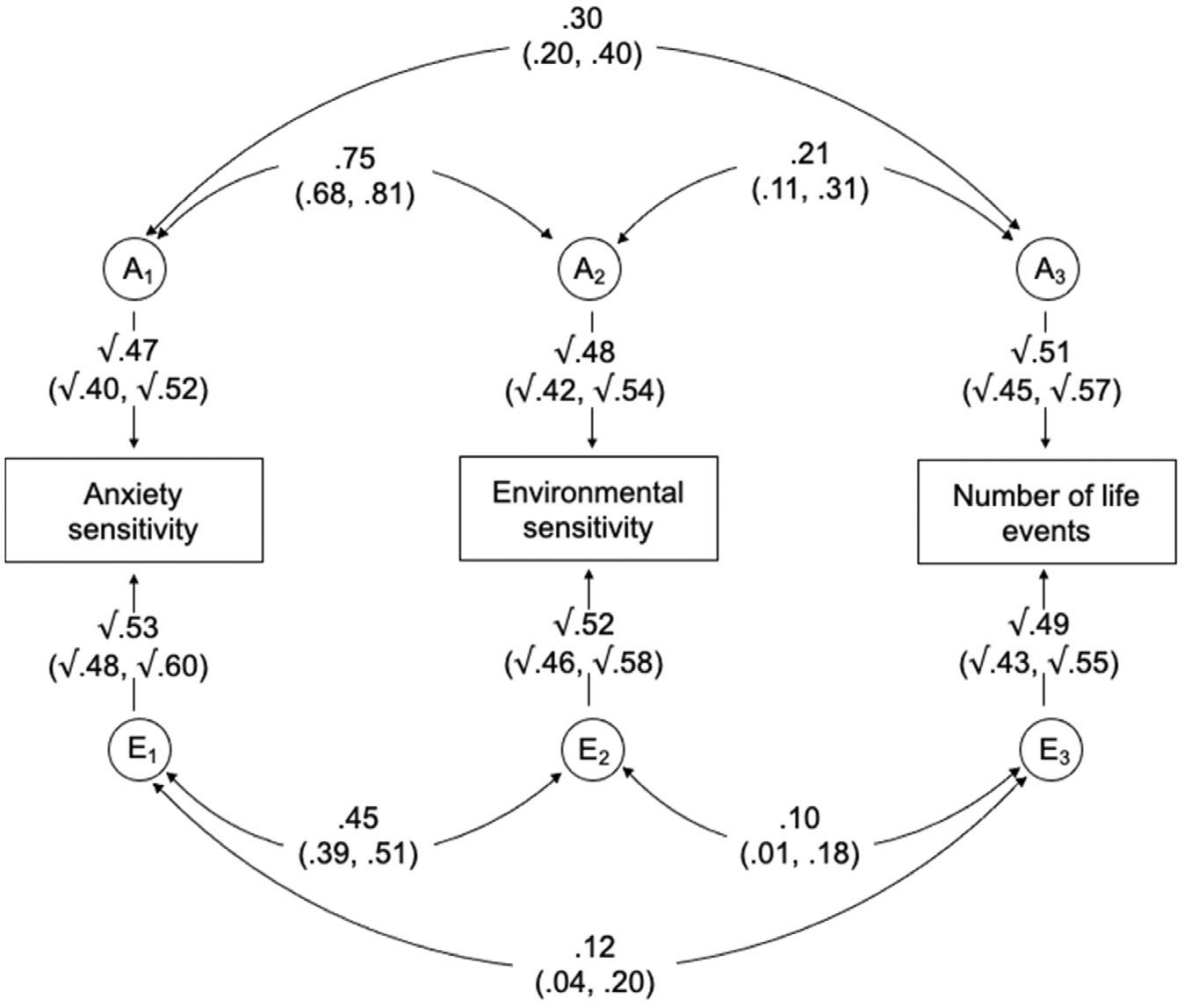
Correlated factors solution of the multivariate Cholesky decomposition for anxiety sensitivity, environmental sensitivity and number of life events. A_1–3_ and E_1–3_ represent the respective additive genetic and nonshared environmental influences (95% CIs). Curved paths show the correlations between the A and E factors for each measure (95% CIs)

**Figure 2:**
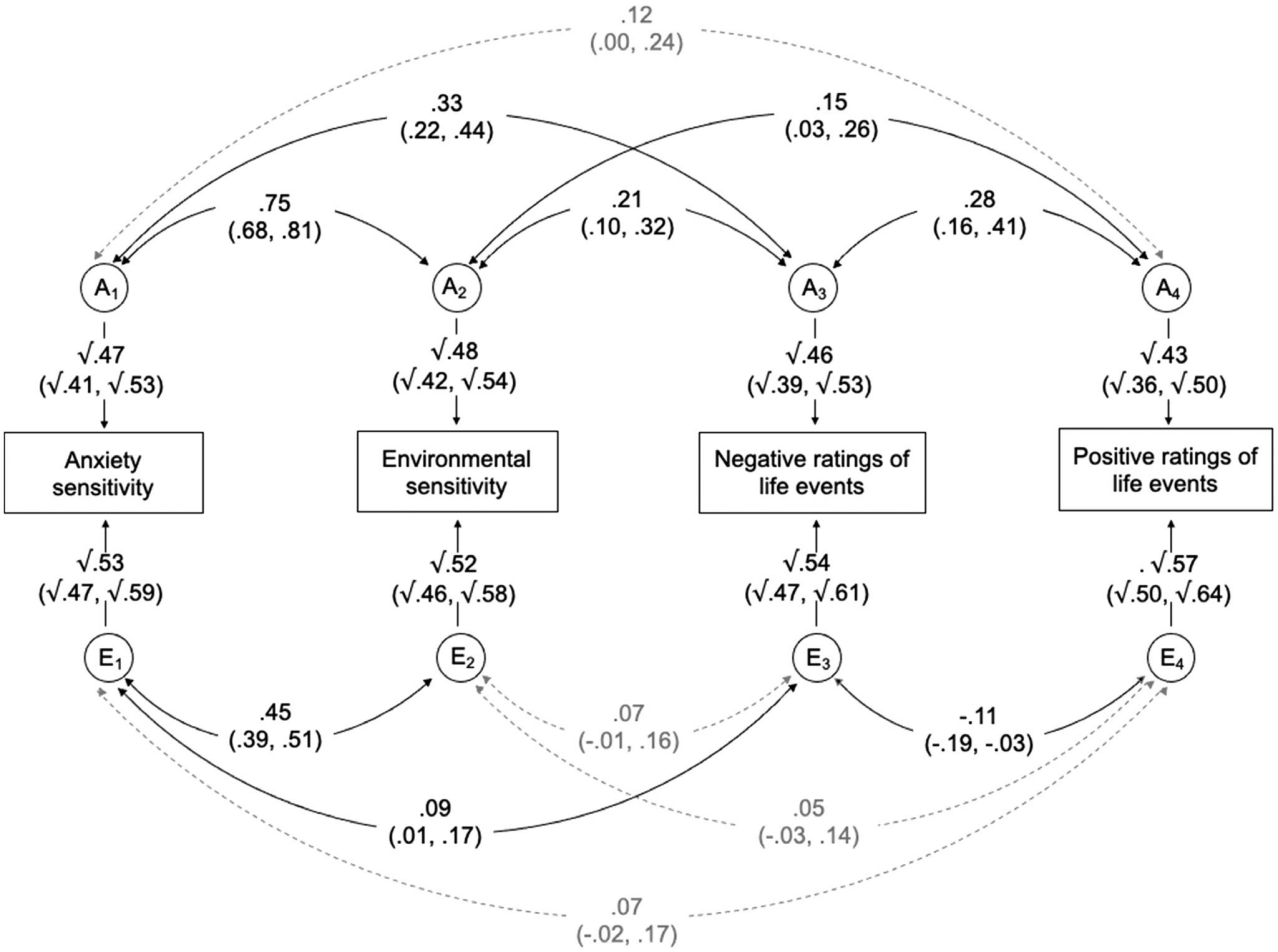
Correlated factors solution of the multivariate Cholesky decomposition for anxiety sensitivity, environmental sensitivity, negative ratings of life events and positive ratings of life events. A_1–4_ and E_1–4_ represent the respective additive genetic and nonshared environmental influences (95% CIs). Curved paths show the correlations between the A and E factors for each measure (95% CIs)

**Figure 3:**
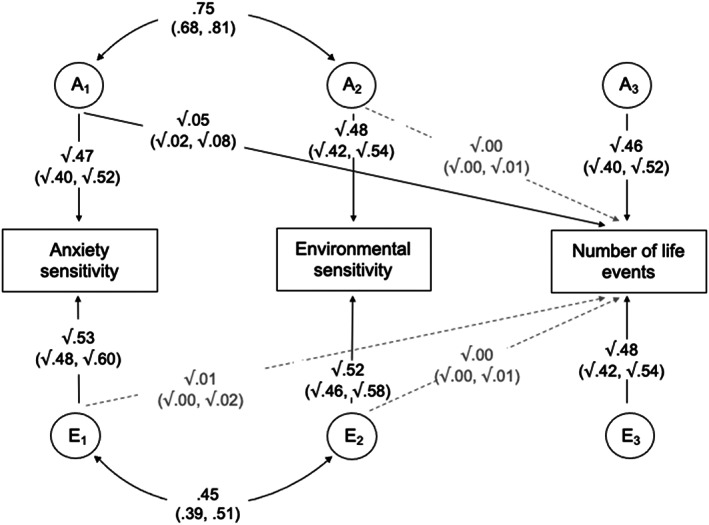
Unique genetic and environmental influences on number of life events, over and above those shared with anxiety sensitivity and environmental sensitivity. A_1–3_ and E_1–3_ represent the respective additive genetic and nonshared environmental influences (95% CIs). Curved paths show the correlation between the A and E factors for anxiety sensitivity and environmental sensitivity (95% CIs)

The proportions of the phenotypic correlation explained by A and E are calculated as, for example $\sqrt{{h^{2}}_{1}}\times r_{A12}\times \sqrt{{h^{2}}_{2}}$ and $\sqrt{{e^{2}}_{1}}\times r_{E12}\times \sqrt{{e^{2}}_{2}}$, where *r*_A12_/*r*_E12_ = coefficients of the curved double‐headed arrows as in Figures 1, 2, 3. These proportions are converted to percentages of the phenotypic correlation by dividing them by *r*_ph_. Estimates based on nonsignificant *r*_A_/*r*_E_s should be interpreted with caution .

### Multivariate models

Comparison of saturated and constrained models indicated that the assumptions of equality of means and variances were met (*p* = .128–.249). Across ACE models, C estimates were generally small (<9%) and nonsignificant. For all models, dropping the C parameters did not result in significant worsening of fit (Models 1 and 3: χ^2^(6) = 2.585057, *p* = .859, Model 2: χ^2^(10) = 6.644232, *p* = .759). Hence, AE models are presented. The full ACE models are provided in Figures S1 and S2. Model fit statistics and comparisons are given in Tables S9 and S10.

Multivariate AE models are presented below and summarised in Table 2. Standardised A and E influences are shown as *h* ^2^ and *e* ^2^. To illustrate associations between measures of sensitivity and life events variables, cross‐twin cross‐trait correlations are given for MZ (*r*_MZ_) and DZ (*r*_DZ_) twin pairs, and within‐person phenotypic correlations (*r*_ph_) are presented with the standardised proportions of the phenotypic associations accounted for by additive genetic (A) and unique environmental (E) influences.

#### Model 1

The Cholesky decomposition, represented as a multivariate correlated factors solution, was used to examine the genetic and environmental relationship between anxiety sensitivity, environmental sensitivity and the number of reported life events (Figure 1).

As hypothesised, both anxiety sensitivity and environmental sensitivity showed moderate genetic and small nonshared environmental correlations with the number of reported life events (*r*_A_ = .30 and .21, *r*_E_ = .12 and .10, respectively). Genetic influences accounted for 70% of the phenotypic correlation between anxiety sensitivity and life events, and 69% for environmental sensitivity and life events (Table 2). Anxiety sensitivity and environmental sensitivity showed a high genetic correlation (*r*_A_ = .75), with genetic influences accounting for 60% of the phenotypic association.

#### Model 2

The multivariate correlated factors solution was extended to examine the shared genetic and environmental influences between the two measures of sensitivity and negative and positive ratings of life events (Figure 2).

Negative ratings of life events showed moderate genetic correlations with both anxiety sensitivity and environmental sensitivity (*r*_A_ = .33 and .21, respectively). Genetic influences accounted for 75% and 72% of the phenotypic correlations, respectively. For positive ratings of life events, genetic correlations with both measures were lower and were only significant for environmental sensitivity (*r*_A_ = .15). Wide confidence intervals around the proportion of covariance attributable to genetic factors indicate that these cannot be accurately inferred.

#### Model 3

A variation of Model 1 was used to investigate the proportion of genetic and environmental influences on reported life events shared with anxiety sensitivity and environmental sensitivity (Figure 3).

After accounting for the genetic effects on both sensitivity measures, unique genetic influences accounted for a smaller proportion of the variance of reported life events, at 46% compared to 51% when anxiety sensitivity and environmental sensitivity were not adjusted for (Model 1).

Hence, 90% (95% CIs 84–96) of the genetic influences on reported life events were independent of the influences on anxiety sensitivity and environmental sensitivity (46%/51% × 100). Therefore, 10% (95% CIs 4–16) of the genetic influences on reported life events were accounted for by the genetic influences on these measures.

## Discussion

The aim of this study was to explore the shared genetic and environmental influences on anxiety sensitivity, environmental sensitivity and reported life events in adolescence. The majority of the phenotypic associations between all measures were explained by shared genetic influences (60%–75%), with the remainder explained by nonshared environmental influences (25%–40%). Environmental sensitivity showed comparable genetic correlations with both negative and positive ratings of life events (*r*_A_ = .21 and .15), whereas anxiety sensitivity only showed a significant genetic correlation with negative ratings of life events (*r*_A_ = .33). Approximately 10% of the heritability of reported life events was accounted for by the genetic influences on anxiety sensitivity and environmental sensitivity.

Consistent with previous research, anxiety sensitivity and environmental sensitivity showed moderate heritability, with the remaining variance explained by nonshared environmental influences (Assary et al., 2021; Zavos et al., 2010). Despite assessing different forms of sensitivity, relating to pathological interpretations of anxiety symptoms and variation in response to environments, the moderate phenotypic correlation between these measures indicates that they represent related constructs. The high genetic contribution to their covariance suggests that this phenotypic similarity is driven by shared genetic influences. For environmental sensitivity, three components have been identified, representing reactivity to sensory stimuli, becoming overwhelmed by contextual emotional psychological stimuli and greater aesthetic appreciation (Smolewska, McCabe, & Woody, 2006). As anxiety sensitivity captures negative interpretation of those responses, it is logical that a large proportion of its genetic influences are shared with the sensitivity to environmental stimuli. Therefore, the present findings suggest that many of the same genetic factors that influence overall response to external stimuli also influence the interpretation of internal responses as harmful.

As expected, life events also displayed moderate heritability (Kendler & Baker, 2007), with covariance between life events and sensitivity measures being predominantly genetically driven. Genetic influences on environmental measures can be indicative of gene–environment correlation, whereby genetic factors influence the environments that individuals are exposed to, either passively through parents, or actively or evocatively through genetically influenced behaviours (Jaffee & Price, 2007). In this context, gene–environment correlation could occur through genetic influences on sensitivity creating a tendency for individuals to seek out or elicit environments that increase the likelihood of experiencing life events (Zavos, Wong, et al., 2012). Under this framework, we would expect that individuals with greater genetic loading for sensitivity would also report greater exposure to life events.

An alternative plausible explanation for genetic overlap is that the genetic factors that influence sensitivity are not correlated with *exposure* to life events, but rather they influence an individual's subjective experience. As life events were assessed using self‐report, this measure is likely to capture individual differences in the interpretation or impact of events, how they are recalled and willingness to disclose personal experiences. These aspects of self‐reporting may share a genetic basis with sensitivity. This explanation is consistent with the relatively low phenotypic correlations between sensitivity and life events (*r*_ph_ = .09–.21), which indicates that expression of these measures share limited overall variance. This may imply that while sensitivity contributes to the subjective experience of an event, as captured by self‐reports, it cannot fully account for differences in exposure to environmental risk. However, the large confidence intervals around the proportions of covariance attributable to genetic factors indicate that these estimates should be interpreted with caution.

The differential pattern of associations between the two types of sensitivity with negative and positive ratings of life events is consistent with the theoretical basis of these measures. The finding that environmental sensitivity was significantly genetically correlated with both positive and negative appraisals may suggest that higher genetic loading for this form of sensitivity is associated with greater appraisal of both adverse events as negative, and pleasant events as positive, which may attenuate the effects of such events on outcomes for these individuals. This is in line with evidence that more sensitive individuals are affected more negatively by adverse contexts but also more positively in response to positive exposures (Pluess et al., 2020). On the other hand, the underlying genetic liability of anxiety sensitivity is more relevant to the appraisal of events as negative and the tendency to perceive anxiety responses to these events as being harmful. This corresponds to previous findings that anxiety sensitivity moderates the association between stressful life events and later internalising symptoms (McLaughlin & Hatzenbuehler, 2009). This suggests that while sensitivity to contextual aspects of the environment may be important to the interpretation of both negative and positive events, sensitivity to one's own anxiety responses plays a greater role in the perception of an environment as adverse. Hence, interventions targeting the interpretation of external environmental and internal stimuli may help to reduce the negative impact of perceived negative events.

Lastly, our findings suggest that a proportion of the heritable component of life events is captured by genetic influences on sensitivity. This indicates that differences in how individuals process contextual or internal signals may be one mechanism through which genetic variation influences the experience of life events and may represent targets for intervening in the impact of adversity. Nonetheless, a substantial proportion of the genetic influences on life events were independent of these sensitivity measures. Other efforts to elucidate the heritable component of environmental experiences indicate that some of these remaining influences are likely to be captured by genetic influences on psychopathology and other related constructs, such as personality factors and cognitive biases, which may influence either exposure to or the subjective experience of environmental events (Peel et al., 2022). This knowledge contributes to understanding of what is captured by the heritable component of measures of environmental experiences, and the potential ways in which these experiences may act as risk factors for poor outcomes. However, it is worth noting that sensitivity biases, as captured using questionnaire measures, may not directly translate to differences in interpretation. An important future avenue for this work is to assess whether findings are replicated using alternative measures of perception biases, such as behavioural tasks.

There are a number of strengths in this study. First, it demonstrates the utility of the twin design in exploring genetic overlap for complex traits. There is considerable variation in the assessment of life events across research studies. This variation results in difficulty obtaining the large, homogeneous samples required for molecular genetic analyses. Where sufficient data are available, there are limitations surrounding sample ascertainment. For example, reports of stressful life events are often collected in large‐scale mental health studies, enabling investigation into the genetic variants associated with adversity in the context of disorder (Power et al., 2013). However, these cohorts are typically enriched for affected individuals, therefore, results are unlikely to reflect the general population (Power et al., 2013). Furthermore, there is a scarcity of measures capturing the experience of positive events, despite these also being relevant to mental health and wellbeing. Although the specific genetic variants underlying sensitivity cannot be detected through the twin design, this analysis provides initial evidence that a proportion of the genetic underpinnings of self‐reported life events are shared with sensitivity in a representative sample of adolescents. Additionally, to our knowledge, this is the first investigation of differential patterns of genetic and environmental influences between sensitivity and negative and positive appraisals of life events. This enabled the investigation of how the theoretical assumptions of anxiety and environmental sensitivity translate at the genetic level.

However, several limitations should also be considered. First, dependent and independent life events were grouped together, despite some evidence indicating that the former display greater heritability (Kendler & Baker, 2007). This decision was driven by difficulty in categorising events based on the assumed influence of an individual's behaviour, which is likely to vary widely. For example, ‘Being hospitalized for illness or injury’ could be either dependent or independent of behaviour. Secondly, the classification of negative and positive events was determined based on each twin's appraisals. This was decided with the aim of capturing individual perception rather than assessing exposure to predetermined negative/positive events, and because appraisals for each event were distributed across the full scale of responses (Table S5). However, this approach limits the conclusions that can be drawn about influences on the reporting of specific types of events. Additionally, the reasons behind a small proportion of participants rating objectively negative events as positive, and vice versa, are unclear and may have increased error in the associations with these variables. As all measures were collected at the same time point, data are cross‐sectional and causality should not be assumed. Although interpretations are primarily given in the direction of sensitivity influencing life events, it is possible that the experience of life events influences the development of sensitivity (Zavos, Wong, et al., 2012). As data on sensitivity and positive life events were only collected in the LEAP‐2 assessment, we were not able to utilise longitudinal data to assess the direction of association in these analyses. Finally, the twin design has some inherent limitations, including the assumption that MZ and DZ twin pairs share their environments to the same degree (Rijsdijk & Sham, 2002).

This study contributes to knowledge of what is captured by the heritable component of self‐reported life events. Due to their ease of application and low cost, self‐reported measures are becoming an increasingly popular method of assessing environmental experiences in large samples. Hence, it is important to investigate the traits that influence their response, to increase understanding of the aspects of experience that are captured by these measures. Our findings indicate that sensitivity biases are among these relevant traits, displaying shared genetic propensity with reporting of life events and appraisal of events as negative or positive. This work reinforces the importance of using genetically sensitive designs when investigating life events as environmental risk factors. This may be especially important to consider when examining associations between reported life events and outcomes related to sensitivity in adolescence, including depression (Waszczuk et al., 2015), anxiety (Zavos, Rijsdijk, & Eley, 2012) and personality traits (Assary et al., 2021), as genetic influences are common across these phenotypes. Furthermore, it demonstrates the need for nuanced interpretation of self‐reported life events as measures that captures elements of both exposure and subjective experience.

The finding that sensitivity biases are among the heritable factors that comprise the genetic component of life events is consistent with growing evidence that the subjective perception of the environment plays an important role in its impact on the individual (Danese & Widom, 2020). Further knowledge of how sensitivity relates to the experience and outcomes of life events could provide novel avenues for mental health interventions in those with high genetic propensity for sensitivity. Given that life events represent a key risk factor for numerous psychiatric disorders, increased understanding of the mechanisms through which reported life events confer risk has the potential for widespread impact. Currently, the majority of research investigating the heritable basis of sensitivity has focussed on the expression of these traits in childhood and adolescence. As the genetic influences on many related constructs are found to increase throughout development, a key avenue for future research is the investigation of these relationships across the lifespan.

## Supporting information


**Table S1.** Comparison of Twins Early Development Study (TEDS) families at age 16 with and without data from the LEAP‐2 booklet (*N* = 9,917).
**Table S2.** Children's Anxiety Sensitivity Index.
**Table S3.** Highly Sensitive Child Questionnaire.
**Table S4.** Coddington Life Events Scale.
**Table S5.** Classification of twin‐specific and family‐wide events from the Coddington Life Events Scale (Coddington, 1972) and proportion of responses for each event (*N* = 2,939).
**Table S6.** Response coding for three variables created from the Coddington Life Events Scale.
**Table S7.** Univariate model fitting results.
**Table S8.** Phenotypic Pearson's correlations between all variables for the full sample and split by sex.
**Table S9.** Model fit statistics for Models 1 and 3 (including anxiety sensitivity, environmental sensitivity and number of reported life events).
**Table S10.** Model fit statistics for Model 2 (including anxiety sensitivity, environmental sensitivity, negative ratings of life events and positive ratings of life events).
**Figure S1.** Full ACE model of anxiety sensitivity, environmental sensitivity and number of life events.
**Figure S2.** Full ACE model of anxiety sensitivity, environmental sensitivity, negative ratings of life events and positive ratings of life events.
