# Supplementary material for: A multivariate genetic analysis of anxiety sensitivity, environmental sensitivity and reported life events in adolescents
Source: J Child Psychol Psychiatry. 2022 Dec 13;64(2):289–98. doi: 10.1111/jcpp.13725 (PMC10107936; doi:10.1111/jcpp.13725)
Supplement: Supplementary file 1 — Table S1. Comparison of Twins Early Development Study (TEDS) families at age 16 with and without data from the LEAP‐2 booklet (N = 9,917). Table S2. Children's Anxiety Sensitivity Index. Table S3. Highly Sensitive Child Questionnaire. Table S4. Coddington Life Events Scale. Table S5. Classification of twin‐specific and family‐wide events from the Coddington Life Events Scale (Coddington, 1972) and proportion of responses for each event (N = 2,939). Table S6. Response coding for three variables created from the Coddington Life Events Scale. Table S7. Univariate model fitting results. Table S8. Phenotypic Pearson's correlations between all variables for the full sample and split by sex. Table S9. Model fit statistics for Models 1 and 3 (including anxiety sensitivity, environmental sensitivity and number of reported life events). Table S10. Model fit statistics for Model 2 (including anxiety sensitivity, environmental sensitivity, negative ratings of life events and positive ratings of life events). Figure S1. Full ACE model of anxiety sensitivity, environmental sensitivity and number of life events. Figure S2. Full ACE model of anxiety sensitivity, environmental sensitivity, negative ratings of life events and positive ratings of life events. [file JCPP-64-289-s001.docx]

**Supporting Information**

**A multivariate genetic analysis of anxiety sensitivity, environmental sensitivity and reported life events in adolescents**

Alicia J. Peel^1(^[^0000-0002-6144-5412^](https://orcid.org/0000-0002-6144-5412)^)^, Olakunle Oginni^1(0000-0002-2340-9429)^,

Elham Assary^1(0000-0001-9788-0478)^, Georgina Krebs^1,2,3(0000-0002-5353-5645)^,

Celestine Lockhart^1(0000-0002-6584-3696)^, Thomas McGregor^1(^[^0000-0003-0024-7049^](https://orcid.org/0000-0003-0024-7049)^)^,

Elisavet Palaiologou^1(0000-0003-4707-3349)^, Angelica Ronald^4(0000-0002-9576-2176)^,

Andrea Danese^1,5,6(^[^0000-0001-8718-5412^](https://orcid.org/0000-0001-8718-5412)^)^ & Thalia C. Eley^1,7(^[^0000-0001-6458-0700^](https://orcid.org/0000-0001-6458-0700)^)^

1. Social, Genetic and Developmental Psychiatry Centre, Institute of Psychiatry, Psychology and Neuroscience, King's College London, UK
2. Research Department of Clinical, Educational and Health Psychology, University College London, UK
3. National and Specialist OCD, BDD and Related Disorders Clinic for Young People, South London and Maudsley NHS Foundation Trust, UK
4. Centre for Brain and Cognitive Development, Department of Psychological Sciences, Birkbeck, University of London, UK
5. Department of Child & Adolescent Psychiatry, Institute of Psychiatry, Psychology & Neuroscience, King's College London, UK
6. National and Specialist CAMHS Trauma, Anxiety, and Depression Clinic, South London and Maudsley NHS Foundation Trust, UK
7. NIHR Biomedical Research Centre, South London and Maudsley Hospital, UK

**Correspondence to**

Thalia Eley, thalia.eley@kcl.ac.uk, 020 7848 0863

Room C1.16, Social, Genetic and Developmental Psychiatry Centre, Institute of Psychiatry, Psychology & Neuroscience, King’s College London, London, United Kingdom. SE5 8AF

**Contents**

[Tables 2](#_Toc119076099)

[**Table S1.** Comparison of Twins Early Development Study (TEDS) families at age 16 with and without data from the LEAP-2 booklet (*N* = 9917) 2](#_Toc119076100)

[**Table S2.** Children's Anxiety Sensitivity Index (Silverman et al., 2013) 3](#_Toc119076101)

[**Table S3.** Highly Sensitive Child Questionnaire (Pluess et al., 2018) 4](#_Toc119076102)

[**Table S4.** Coddington Life Events Scale (Coddington, 1972) 5](#_Toc119076103)

[**Table S5.** Classification of twin-specific and family-wide events from the Coddington Life Events Scale (Coddington, 1972) and proportion of responses for each event (*N* = 2939) 7](#_Toc119076104)

[**Table S6.** Response coding for three variables created from the Coddington Life Events Scale (Coddington, 1972) 9](#_Toc119076105)

[**Table S7.** Univariate model fitting results 10](#_Toc119076106)

[**Table S8.** Phenotypic Pearson’s correlations between all variables for the full sample and split by sex 11](#_Toc119076107)

[**Table S9.** Model fit statistics for Models 1 and 3 (including anxiety sensitivity, environmental sensitivity and number of reported life events) 12](#_Toc119076108)

[**Table S10.** Model fit statistics for Model 2 (including anxiety sensitivity, environmental sensitivity, negative ratings of life events and positive ratings of life events) 13](#_Toc119076109)

[Figures 14](#_Toc119076110)

[**Figure S1.** Full ACE model of anxiety sensitivity, environmental sensitivity and number of life events 14](#_Toc119076111)

[**Figure S2.** Full ACE model of anxiety sensitivity, environmental sensitivity, negative ratings of life events and positive ratings of life events 15](#_Toc119076112)

# Tables

##

## **Table S1.** Comparison of Twins Early Development Study (TEDS) families at age 16 with and without data from the LEAP-2 booklet (*N* = 9917)

|  | **Families with LEAP-2 data (*n* = 2943)** | **Families without LEAP-2 data**  **(*n* = 6974)** | **Comparison of families with and without LEAP-2 data (total *N* from age 16 LEAP Study = 9917)** |
| --- | --- | --- | --- |
| White ethnicity | 92.5% | 93.9% | χ^2^(1) = 5.2404, *p* = .022, *V* = .02* |
| Mothers with A-levels or higher | 24.0% | 22.7% | χ^2^(1) = 0.0001, *p* = .992, *V* = .00 |
| Fathers with A-levels or higher | 19.8% | 20.1% | χ^2^(1) = 0.9345, *p* = .334, *V* = .02 |
| Female | 57.8% | 54.6% | χ^2^(1) = 8.1743, *p* = .004, *V* = .03* |
| MZ | 35.2% | 36.6% | χ^2^(1) = 1.7094, *p* = .191, *V* = .01 |
| Reported at least one life event in LEAP Study | 75.0% | 76.1% | χ^2^(1) = 2.1104, *p* = .146, *V* = .02 |

*Notes:* * = difference significant at *p* < 0.05. Selection for LEAP-2 consisted of families in which one or both twins had scored highly in measures of psychotic experiences in the LEAP study (1420 pairs) and 'control' families in which neither twin had high scores in these measures (355 pairs). Of the families invited, 1,471 (83%) returned the LEAP-2 booklets. Families with LEAP-2 data were more ethnically diverse and included a higher proportion of female twins than those who were not invited to or did not complete the LEAP-2 study, but they did not differ on family socioeconomic characteristics or in the proportion of twins who reported a life event.

## **Table S2.** Children's Anxiety Sensitivity Index (Silverman et al., 2013)

| **How true are the following statements when you think about your feelings over the last six months?** |
| --- |

|  | Not true | Quite true | Very true |
| --- | --- | --- | --- |
|  | 0 | 1 | 2 |
| 1. I don't want other people to know when I feel afraid |  |  |  |
| 2. When I cannot keep my mind on my schoolwork, I worry that I might be going crazy |  |  |  |
| 3. It scares me when I feel "shaky" |  |  |  |
| 4. It scares me when I feel like I am going to faint |  |  |  |
| 5. It is important for me to stay in control of my feelings |  |  |  |
| 6. It scares me when my heart beats fast |  |  |  |
| 7. I feel embarrassed when my stomach rumbles or makes noise |  |  |  |
| 8. It scares me when I feel like I am going to throw up |  |  |  |
| 9. When I notice that my heart is beating fast, I worry that there might be something wrong with me |  |  |  |
| 10. It scares me when I have trouble getting my breath |  |  |  |
| 11. When my stomach hurts, I worry that I might be really ill |  |  |  |
| 12. It scares me when I cannot concentrate on my schoolwork |  |  |  |
| 13. Others my age can tell when I feel shaky |  |  |  |
| 14. Unusual feelings in my body scare me |  |  |  |
| 15. When I am afraid, I worry that I might be crazy |  |  |  |
| 16. I get scared when I feel nervous |  |  |  |
| 17. I don't like to let my feelings show |  |  |  |
| 18. Funny feelings in my body scare me |  |  |  |

## **Table S3.** Highly Sensitive Child Questionnaire (Pluess et al., 2018)

| **For each of the following statements, please select the point on the scale that you feel is most appropriate in describing you.** |
| --- |

|  | Not at all | |  |  | Moderately | | |  |  | Extremely | |
| --- | --- | --- | --- | --- | --- | --- | --- | --- | --- | --- | --- |
|  | 1 | 2 | | 3 | | 4 | 5 | | 6 | | 7 |
| 1. I notice when small things have changed in my environment |  |  | |  | |  |  | |  | |  |
| 2. Loud noises make me feel uncomfortable |  |  | |  | |  |  | |  | |  |
| 3. I love nice smells |  |  | |  | |  |  | |  | |  |
| 4. I get nervous when I have to do a lot in little time |  |  | |  | |  |  | |  | |  |
| 5. Some music can make me really happy |  |  | |  | |  |  | |  | |  |
| 6. I am annoyed when people try to get me to do too many things at once |  |  | |  | |  |  | |  | |  |
| 7. I don’t like watching TV programmes that have a lot of violence in them |  |  | |  | |  |  | |  | |  |
| 8. I find it unpleasant to have a lot going on at once |  |  | |  | |  |  | |  | |  |
| 9. I don’t like it when things change in my life |  |  | |  | |  |  | |  | |  |
| 10. I love nice tastes |  |  | |  | |  |  | |  | |  |
| 11. I don’t like loud noises |  |  | |  | |  |  | |  | |  |
| 12. When someone observes me, I get nervous. This makes me perform worse than normal |  |  | |  | |  |  | |  | |  |

## **Table S4.** Coddington Life Events Scale (Coddington, 1972)

| **Here is a list of events that might have happened to you recently. Please put a tick in either the ‘No’ or ‘Yes’ box to say whether the event has happened in the past six months. If you answer ‘yes’ then please indicate what it was like, choosing one of the options given, ranging from ‘very unpleasant’ to ‘very pleasant’.**  In the past six months, I have experienced... |
| --- |

|  | Yes | No | Very unpleasant | Moderately  unpleasant | Neither unpleasant nor pleasant | Moderately pleasant | Very pleasant |
| --- | --- | --- | --- | --- | --- | --- | --- |
| 1. The loss of a job by my father or mother |  |  |  |  |  |  |  |
| 2. Marital separation of my parents |  |  |  |  |  |  |  |
| 3. Becoming involved with drugs |  |  |  |  |  |  |  |
| 4. The death of a close friend or relative |  |  |  |  |  |  |  |
| 5. Being hospitalized for illness or injury |  |  |  |  |  |  |  |
| 6. Being sent away from home |  |  |  |  |  |  |  |
| 7. Breaking up with a boyfriend/girlfriend |  |  |  |  |  |  |  |
| 8. The hospitalization of my brother or sister |  |  |  |  |  |  |  |
| 9. Suspension from school/college |  |  |  |  |  |  |  |
| 10. Failing an important exam |  |  |  |  |  |  |  |
| 11. Remarriage of a parent to a stepparent |  |  |  |  |  |  |  |
| 12. Hospitalization of a parent |  |  |  |  |  |  |  |
| 13. Being responsible for a road accident |  |  |  |  |  |  |  |
| 14. A major decrease in parental income |  |  |  |  |  |  |  |
| 15. Getting pregnant or fathering a pregnancy |  |  |  |  |  |  |  |
| 16. Outstanding personal achievement |  |  |  |  |  |  |  |
| 17. Decrease in number of arguments between parents |  |  |  |  |  |  |  |
| 18. Becoming a member of a church |  |  |  |  |  |  |  |
| 19. Beginning to date |  |  |  |  |  |  |  |
| 20. Moving to a new school or college |  |  |  |  |  |  |  |

## **Table S5.** Classification of twin-specific and family-wide events from the Coddington Life Events Scale (Coddington, 1972) and proportion of responses for each event (*N* = 2939)

|  | **Number of responses (%)** | | | | | | | | |
| --- | --- | --- | --- | --- | --- | --- | --- | --- | --- |
|  | No | Very unpleasant | | Moderately  unpleasant | Neither unpleasant nor pleasant | Moderately pleasant | Very pleasant | | NA |
| **Family-wide events** | | | | | | | | | |
| The loss of a job by my father or mother | 2704 (92.0%) | | 34  (1.2%) | 76  (2.6%) | 52  (1.8%) | 3  (0.1%) | 2  (0.1%) | 68  (2.3%) | |
| Marital separation of my parents | 2820 (96.0%) | | 28  (1.0%) | 20  (0.7%) | 13  (0.4%) | 0 | 1  (0.0%) | 57  (1.9%) | |
| The death of a close friend or relative | 2479 (84.4%) | | 270  (9.2%) | 111  (3.8%) | 19  (0.7%) | 5  (0.2%) | 3  (0.1%) | 52  (1.8%) | |
| The hospitalization of my brother or sister | 2705 (92.0%) | | 37  (1.3%) | 53  (1.8%) | 20  (0.7%) | 1  (0.0%) | 0 | 123  (4.2%) | |
| Remarriage of a parent to a stepparent | 2847 (96.9%) | | 8  (0.3%) | 8  (0.3%) | 4  (0.1%) | 11  (0.4%) | 6  (0.2%) | 55  (1.9%) | |
| Hospitalization of a parent | 2721 (92.6%) | | 85  (2.9%) | 65  (2.2%) | 19  (0.6%) | 1  (0.0%) | 2  (0.1%) | 46  (1.6%) | |
| A major decrease in parental income | 2666 (90.7%) | | 79  (2.7%) | 102  (3.5%) | 38  (1.3%) | 1  (0.0%) | 0 | 53  (1.8%) | |
| Decrease in number of arguments between parents | 2277 (77.5%) | | 25  (0.9%) | 21  (0.7%) | 40  (1.4%) | 221  (7.5%) | 246  (8.4%) | 109  (3.7%) | |
| **Twin-specific events** | | | | | | | |  | |
| Becoming involved with drugs | 2751 (93.6%) | | 6  (0.2%) | 9  (0.3%) | 42  (1.4%) | 51  (1.7%) | 12  (0.4%) | 68  (2.3%) | |
| Being hospitalized for illness or injury | 2736 (93.1%) | | 46  (1.6%) | 62  (2.1%) | 33  (1.1%) | 5  (0.2%) | 3  (0.1%) | 54  (1.8%) | |
| Being sent away from home | 2800 (95.3%) | | 6  (0.2%) | 20  (0.7%) | 14  (0.5%) | 23  (0.8%) | 17  (0.6%) | 59  (2.0%) | |
| Breaking up with a boyfriend/girlfriend | 2463 (83.8%) | | 191  (6.5%) | 145  (4.9%) | 49  (1.7%) | 16  (0.5%) | 11  (0.4%) | 64  (2.2%) | |
| Suspension from school/college | 2850 (97.0%) | | 9  (0.3%) | 15  (0.5%) | 12  (0.4%) | 3  (0.1%) | 6  (0.2%) | 44  (1.5%) | |
| Failing an important exam | 2297 (78.2%) | | 344  (11.7%) | 184  (6.3%) | 46  (1.6%) | 2  (0.1%) | 4  (0.1%) | 62  (2.1%) | |
| Being responsible for a road accident | 2878 (97.9%) | | 4  (0.1%) | 6  (0.2%) | 3  (0.1%) | 0 | 2  (0.1%) | 46  (1.6%) | |
| Getting pregnant or fathering a pregnancy | 2863 (97.4%) | | 10  (0.4%) | 4  (0.1%) | 7  (0.2%) | 3  (0.1%) | 3  (0.1%) | 49  (1.7%) | |
| Outstanding personal achievement | 1864 (63.4%) | | 44  (1.5%) | 15  (0.5%) | 29  (1.0%) | 252  (8.6%) | 662  (22.5%) | 73  (2.5%) | |
| Becoming a member of a church | 2839 (96.6%) | | 6  (0.2%) | 2  (0.1%) | 6  (0.2%) | 14  (0.5%) | 14  (0.5%) | 58  (2.0%) | |
| Beginning to date | 2449 (83.3%) | | 14  (0.5%) | 18  (0.6%) | 33  (1.1%) | 130  (4.4%) | 228  (7.8%) | 67  (2.3%) | |
| Moving to a new school or college | 2548 (86.7%) | | 18  (0.6%) | 50  (1.7%) | 83  (2.8%) | 109  (3.7%) | 78  (2.7%) | 53  (1.8%) | |

*Notes:* Of the 20 items, 8 are considered to be ‘family-wide’, for example ‘Marital separation of my parents’, as they relate to the general family environment. The remaining 12 items are considered to be ‘twin-specific’, for example ‘Failing an important exam’. Both types of events were retained in this analysis, as family-wide experiences can be differentially perceived by individual siblings [(Daniels et al., 1985)](https://paperpile.com/c/hEMaHG/rAbIo) and the correlation between twins on the total number of reported family-wide events was only moderate (ρ = 0.43).

Daniels, D., Dunn, J., Furstenberg, F.F., Jr, & Plomin, R. (1985). Environmental differences within the family and adjustment differences within pairs of adolescent siblings. *Child Development*, *56*(3), 764–774.

## **Table S6.** Response coding for three variables created from the Coddington Life Events Scale (Coddington, 1972)

| **Variable** | **Response coding** | | | | | | |
| --- | --- | --- | --- | --- | --- | --- | --- |
|  | No | Yes | Very unpleasant | Moderately unpleasant | Neither unpleasant nor pleasant | Moderately pleasant | Very pleasant |
| **Total number of life events** | 0 | 1 | 1 | 1 | 1 | 1 | 1 |
| **Positive ratings of events** | 0 | 0 | 0 | 0 | 0 | 1 | 2 |
| **Negative ratings of events** | 0 | 0 | 2 | 1 | 0 | 0 | 0 |

## **Table S7.** Univariate model fitting results

| **Measure** | **ACE variance components (95% CI)** | | |
| --- | --- | --- | --- |
|  | **A** | **C** | **E** |
| Anxiety sensitivity | .46 (.39-.52) | .00 (.00-.04) | .54 (.48-.60) |
| Environmental sensitivity | .48 (.35-.54) | .00 (.00-.09) | .52 (.46-.58) |
| Number of life events | .45 (.26-.56) | .05 (.00-.19) | .50 (.44-.57) |
| Negative ratings of life events | .46 (.31-.52) | .00 (.00-.10) | .54 (.48-.61) |
| Positive ratings of life events | .29 (.09-.49) | .12 (.00-.27) | .69 (.52-.67) |

## **Table S8.** Phenotypic Pearson’s correlations between all variables for the full sample and split by sex

|  | **Full sample**  **(*N* = 2939)** | **Males**  **(*n* = 1239)** | **Females**  **(*n* = 1700)** |
| --- | --- | --- | --- |
| Anxiety sensitivity - Environmental sensitivity | .59 (.57-.62) | .60 (.56-.63) | .60 (.57-.63) |
| Anxiety sensitivity - Number of life events | .20 (.16-.24) | .23 (.17-.28) | .20 (.15-.25) |
| Anxiety sensitivity - Negative ratings of life events | .19 (.15-.23) | .24 (.18-.29) | .16 (.11-.21) |
| Anxiety sensitivity - Positive ratings of life events | .09 (.05-.13) | .12 (.06-.18) | .09 (.04-.15) |
| Environmental sensitivity - Number of life events | .15 (.11-.19) | .13 (.07-.19) | .16 (.11-.21) |
| Environmental sensitivity - Negative ratings of life events | .13 (.09-.17) | .15 (.08-.21) | .14 (.08-.19) |
| Environmental sensitivity - Positive ratings of life events | .10 (.06-.14) | .09 (.03-.15) | .10 (.05-.16) |
| Negative ratings of life event - Positive ratings of life events | .07 (.03-.11) | .10 (.04-.16) | .12 (.07-.17) |

##

## **Table S9.** Model fit statistics for Models 1 and 3 (including anxiety sensitivity, environmental sensitivity and number of reported life events)

| **Base model** | **Comparison** | **−2LL** | **df** | **AIC** | **Δ-2LL** | **Δdf** | **ΔAIC** | ***p*** |
| --- | --- | --- | --- | --- | --- | --- | --- | --- |
| Saturated |  | 34973.03 | 8161 | 35081.03 |  |  |  |  |
| Saturated | Constrained | 35011.12 | 8194 | 35053.12 | 38.09378 | 33 | -27.91 | .249 |
| Saturated | ACE | 35019.47 | 8194 | 35061.47 | 46.4404 | 33 | -19.56 | .060 |
| Constrained | ACE | 35019.47 | 8194 | 35061.47 | 8.346612 | 0 | 8.35 | - |
| Saturated | **AE** | **35022.05** | **8200** | **35052.05** | 49.02545 | 39 | -28.98 | .130 |
| Constrained | **AE** | **35022.05** | **8200** | **35052.05** | 10.93167 | 6 | -1.07 | .091 |
| ACE | **AE** | **35022.05** | **8200** | **35052.05** | 2.585057 | 6 | -9.42 | .859 |

*Notes*: Saturated = model with the maximum number of parameters to get a baseline index of fit; Constrained = submodel of Saturated model with means and variances constrained to be equal across twin order and zygosity, to test the assumptions of the twin design; ACE = model with A, C and E parameters representing genetic, shared environment and non-shared environment influences; AE = submodel where C parameters are fixed to 0; -2LL = minus twice the log likelihood; df = degrees of freedom; AIC Akaike’s information criterion; Δ-2LL = difference in -2LL value; Δ df= difference in degrees of freedom; ΔAIC= difference in AIC value; *p* = *p*-value of Δ-2LL. The ACE model and was a good fit to the data as compared to the saturated model (*p* = .060). Bold typeface indicates final model selected.

## **Table S10.** Model fit statistics for Model 2 (including anxiety sensitivity, environmental sensitivity, negative ratings of life events and positive ratings of life events)

| **Base model** | **Comparison** | **−2LL** | **df** | **AIC** | **Δ-2LL** | **Δdf** | **ΔAIC** | ***p*** |
| --- | --- | --- | --- | --- | --- | --- | --- | --- |
| Saturated |  | 41508.10 | 10476 | 41684.10 |  |  |  |  |
| Saturated | Constrained | 41574.04 | 10530 | 41642.04 | 65.94461 | 54 | -42.06 | .128 |
| Saturated | ACE | 41586.45 | 10530 | 41654.45 | 78.35471 | 54 | -29.65 | .017 |
| Constrained | ACE | 41586.45 | 10530 | 41654.45 | 12.41011 | 0 | 12.41 | - |
| Saturated | **AE** | **41593.10** | **10540** | **41641.10** | 84.99895 | 64 | -43.00 | .041 |
| Constrained | **AE** | **41593.10** | **10540** | **41641.10** | 19.05434 | 10 | -0.94 | .040 |
| ACE | **AE** | **41593.10** | **10540** | **41641.10** | 6.644232 | 10 | -13.35 | .759 |

#

*Notes*: Saturated = model with the maximum number of parameters to get a baseline index of fit; Constrained = submodel of Saturated model with means and variances constrained to be equal across twin order and zygosity, to test the assumptions of the twin design; ACE = model with A, C and E parameters representing genetic, shared environment and non-shared environment influences; AE = submodel where C parameters are fixed to 0; -2LL = minus twice the log likelihood; df = degrees of freedom; AIC Akaike’s information criterion; Δ-2LL = difference in -2LL value; Δ df= difference in degrees of freedom; ΔAIC= difference in AIC value; *p* = *p*-value of Δ-2LL. The ACE model did not fit the data as well as the saturated model (*p* = .017). This can occur in studies with large sample sizes as minimal variance differences between groups can be highly statistically significant. The significant difference between the ACE and saturated model may reflect differences in the variance component influences indicated by the data and as specified in the genetic model. Bold typeface indicates final model selected.

# Figures

##

## **Figure S1.** Full ACE model of anxiety sensitivity, environmental sensitivity and number of life events


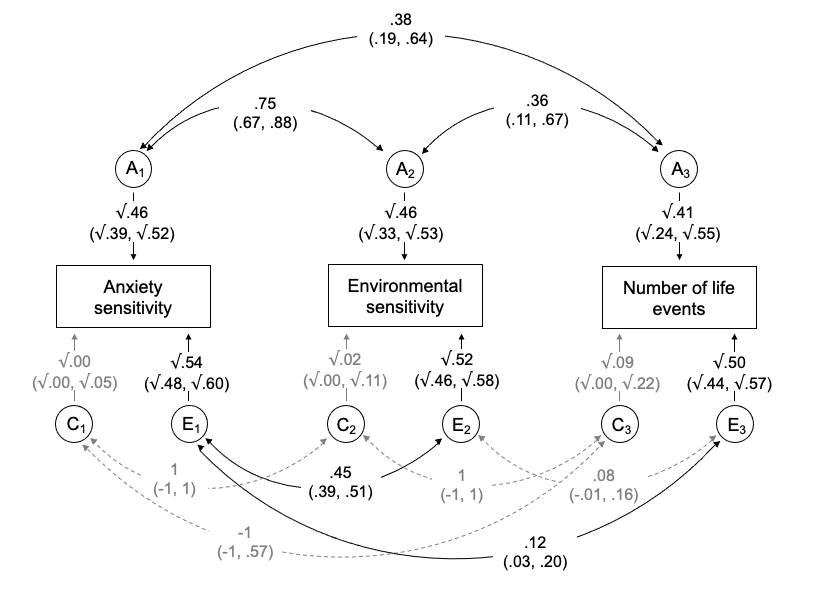


*Figure S1.* Correlated factors solution of the multivariate Cholesky decomposition for anxiety sensitivity, environmental sensitivity and number of life events. A_1-3_, C_1-3_ and E_1-3_ represent the respective additive genetic, shared environmental, and non-shared environmental influences (95% CIs). Curved paths show the correlations between the A, C and E factors for each measure (95% CIs).

## **Figure S2.** Full ACE model of anxiety sensitivity, environmental sensitivity, negative ratings of life events and positive ratings of life events

**
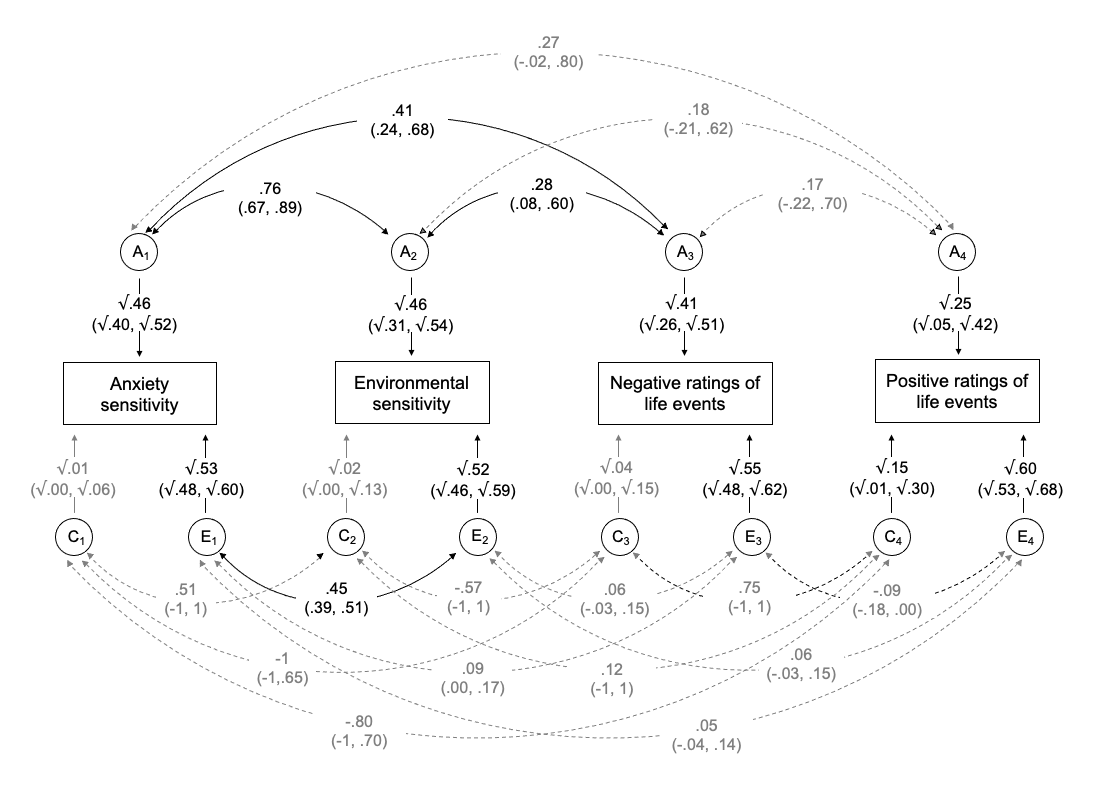
**

*Figure S2.* Correlated factors solution of the multivariate Cholesky decomposition for anxiety sensitivity, environmental sensitivity, negative ratings of life events and positive ratings of life events. A_1-4,_ C_1-4_ and E_1-4_ represent the respective additive genetic, shared environmental and non-shared environmental influences (95% CIs). Curved paths show the correlations between the A, C and E factors for each measure (95% CIs).
